# The dynamics of carbon stored in xylem sapwood to drought-induced hydraulic stress in mature trees

**DOI:** 10.1038/srep24513

**Published:** 2016-04-15

**Authors:** Kenichi Yoshimura, Shin-Taro Saiki, Kenichi Yazaki, Mayumi Y. Ogasa, Makoto Shirai, Takashi Nakano, Jin Yoshimura, Atsushi Ishida

**Affiliations:** 1 Kansai Research Center, Forestry and Forest Products Research Institute, Fushimi, Kyoto 612-0855, Japan; 2 Center for Ecological Research, Kyoto University, Otsu, Shiga 520-2113, Japan; 3 Forestry and Forest Products Research Institute, Tsukuba, Ibaraki 305-8687, Japan; 4 Graduate School of Bioresource Sciences, Nihon University, Fujisawa, Kanagawa 252-0880, Japan; 5 Mount Fuji Research Institute, Yamanashi Prefectural Government. Fuji-Yoshida, Yamanashi 403-0005, Japan; 6 Department of Mathematical and Systems Engineering, Graduate School of Science and Technology, Shizuoka University, Hamamatsu, Shizuoka 432-8561, Japan; 7 Marine Biosystems Research Center, Chiba University, Kamogawa, Chiba 299-5502, Japan; 8 Department of Environmental and Forest Biology, State University of New York College of Environmental Science and Forestry, Syracuse, NY13210, USA

## Abstract

Climate-induced forest die-off is widespread in multiple biomes, strongly affecting the species composition, function and primary production in forest ecosystems. Hydraulic failure and carbon starvation in xylem sapwood are major hypotheses to explain drought-induced tree mortality. Because it is difficult to obtain enough field observations on drought-induced mortality in adult trees, the current understanding of the physiological mechanisms for tree die-offs is still controversial. However, the simultaneous examination of water and carbon uses throughout dehydration and rehydration processes in adult trees will contribute to clarify the roles of hydraulic failure and carbon starvation in tree wilting. Here we show the processes of the percent loss of hydraulic conductivity (PLC) and the content of nonstructural carbohydrates (NSCs) of distal branches in woody plants with contrasting water use strategy. Starch was converted to soluble sugar during PLC progression under drought, and the hydraulic conductivity recovered following water supply. The conversion of NSCs is strongly associated with PLC variations during dehydration and rehydration processes, indicating that stored carbon contributes to tree survival under drought; further carbon starvation can advance hydraulic failure. We predict that even slow-progressing drought degrades forest ecosystems via carbon starvation, causing more frequent catastrophic forest die-offs than the present projection.

Recently, climate-induced forest die-offs have been found world-wide in response to prolonged drought and extreme heating^1^,^2^,^3^,^4^,^5^. Some of the most severe impacts of climate change will be the degradation of forest ecosystems, reducing the ecological benefits. Predictions of forest degradation under future climates are limited by our recent understanding of how mature trees respond to drought and how their survival or die-off is determined under prolonged drought. Currently, two non-pathogen-related hypotheses concerning drought-induced tree mortality have been proposed: hydraulic failure^6^ and carbon starvation^7^,^8^. The hydraulic failure hypothesis states that the physiological process of xylem embolism is caused by prolonged drought and is the major cause of tree and forest die-offs^6^,^9^,^10^,^11^. The carbon starvation hypothesis states that carbon shortages during drought cause physiological dysfunction and thereby lead to the death of forest trees^7^,^12^. Because each hypothesis may be too simple to explain tree mortality^13^,^14^, we sought to collect direct evidence relating to water and carbon use during survival or death processes.

Some studies have proposed that embolized xylem vessels can recover via refilling^15^,^16^,^17^,^18^,^19^ in a process promoted by injection of soluble sugar into the embolized vessels^16^,^20^,^21^,^22^,^23^,^24^,^25^. Experimental studies on seedlings have indicated that an increase in nonstructural carbohydrates (NSCs) in plant bodies enhances their drought tolerance^12^,^16^,^25^. However, the contribution of NSCs to drought tolerance in mature trees remains controversial^26^,^27^, because studies monitoring variations in NSCs and xylem embolism over time are lacking. Leaf photosynthetic and respiration rates and NSCs content in sapwood have been reported to decrease during dehydration processes^28^,^29^, whereas there are no reports of such changes during rehydration processes. Evaluation of carbon starvation and hydraulic failure hypotheses requires monitoring both of whole-plant carbon dynamics and hydraulic dynamics of xylem embolism and recovery during the dehydration and rehydration processes.

To evaluate the relationship between the hydraulic failure and carbon use processes, we simultaneously measured daily time courses of the PLC (percent loss of conductivity) and NSCs content in the xylem sapwood of distal branches, in addition to the leaf water potential, leaf gas exchange and stem respiration rates, in drought-tolerant woody plants. The measurements were periodically conducted during the gradual progression of drought and during the successive hydraulic recovery induced by irrigation or rainfall. To clarify the contribution of NSCs to hydraulic failure, we selected field-growing adult trees of two drought-tolerant woody plants, *Hibiscus glaber* Matsum. and *Ligustrum micranthum* Zucc. (Fig. 1). These species are endemic trees in the Bonin Islands in Japan and are usually found at dry sites with shallow soil^30^. Our data show that they have different mortality rates in distal branches under normal conditions; the longevity of 50% of the distal branches in the same age cohort is 2.4 years in *Hibiscus* and 4.1 years in *Ligustrum,* according to the lower wood density in *Hibiscus* (0.669 g cm^−3^) than *Ligustrum* (0.795 g cm^−3^). Furthermore, xylem cavitation tolerance against dehydration in *Hibiscus* branches is lower than that in *Ligustrum* branches. These interspecific differences should reflect different physiological performance with respect to hydraulic failure and carbon use. Our field study indicates that carbon starvation advances hydraulic failure during slow tree-dieback processes. We discuss the implications of our findings for global climate-induced forest die-offs and their influence on terrestrial ecosystems.

## Results

From the start of the experiment on 22 June 2014, the soil water content gradually decreased except in the case of one very small rainfall event on 11 July (the small shower did not show significant effects on the rehydration of plants). The water content increased rapidly after the areas received a plentiful amount of water (either by irrigation on 15–16 July or by rainfall on 21–22 July) (Fig. 2A). The predawn leaf water potential (*ψ*_pre_) in *Ligustrum* decreased progressively with soil desiccation but increased rapidly following the increase in available water (Fig. 2B). Compared with that in *Ligustrum*, the range of decrease in *ψ*_pre_ in *Hibiscus* was much smaller. Midday net photosynthetic rates and daily stem respiration rates also slightly decreased with soil desiccation (Fig. 2C,D). After water had been provided, the leaf water potential, net photosynthetic rates and stem respiration rates increased in all individuals.

In both species, the PLC in distal branches increased with soil desiccation and decreased within two days of water supply (Fig. 3A,B). Although xylem embolism and hydraulic recovery involve different processes, hysteresis in PLC was not observed between the dehydration and rehydration processes (Fig. 3C,D). At a given *ψ*_pre_, the PLC in *Hibiscus* was higher than that in *Ligustrum*, indicating that the mechanism for escaping severe xylem embolism functions well in *Ligustrum*.

The amounts of soluble sugar and starch varied greatly, and the time-course data from both tree species exhibited a hysteresis loop during the desiccation and rehydration processes (Fig. 4). As PLC increased during soil desiccation, soluble sugar content also increased, and the starch content decreased. After water had been supplied, the soluble sugar content decreased, and the starch content increased. Time-course variations in the NSCs content in *Hibiscus* were greater than in *Ligustrum*. In *Hibiscus*, the change rates in xylem sugar contents were significantly correlated to PLC (*R*^2^ = 0.339, *P* = 0.047) and those in xylem starch contents were insignificantly but nearly correlated to PLC (*R*^2^ = 0.254, *P* = 0.095; Table S1); in *Ligustrum*, those in xylem sugar contents (*R*^2^ = 0.104, *P* = 0.306) and xylem starch contents (*R*^2^ = 0.145, *P* = 0.223) were not correlated to PLC. However, in *Ligustrum*, the amount of xylem sugar contents were insignificantly but nearly correlated to PLC (*R*^2^ = 0.233, *P* = 0.081) and the amount of xylem starch contents significantly decreased with PLC (*R*^2^ = 0.490, *P* = 0.005). Responses of NSCs dynamics in xylem sapwood to water condition were varied for species. Over all, the dynamics of NSCs within xylem sapwood seems to be associated with xylem cavitation (i.e. PLC) rather than water potential.

Soluble sugar content was negatively correlated with starch content during all processes in *Hibiscus* and during the desiccation process in *Ligustrum* (Fig. 5). These relationships did not exhibit hysteresis during the dehydration and rehydration processes. Xylem cavitation triggers starch-to-sugar conversion, whereas the refilling of the embolized vessels promotes the reverse conversion. *Hibiscus* trees exhibited higher starch content and larger PLC variations than *Ligustrum* (Figs 4 and 5). The relatively small PLC variations in *Ligustrum* may explain the reason for non-significant correlation during the rehydration process in *Ligustrum* (Fig. 5B).

## Discussion

In the present study, we show that PLC can be a trigger of carbon dynamics between starch and soluble sugars within xylem sapwood in adult trees, and the amount of carbon dynamically transformed varies among tree species. The differences between the two species used in our study illustrate well that the carbon stored in xylem sapwood is an important determinant of the survival or drought-induced mortality of branches. During soil desiccation and watering, the distal branches of *Hibiscus* showed a greater change in PLC, with larger amounts of soluble sugar investment, than did the branches of *Ligustrum* (Fig. 4). These results are consistent with the physiology and anatomy of these trees; that is, healthy *Hibiscus* trees have a high NSCs (Fig. 5) and a larger fraction of parenchyma cells in their sapwood^30^. The short lifespan of *Hibiscus* twigs would be due to a high critical threshold of NSCs for shoot survival, and the frequent hydraulic segmentation of branches would function to suppress the transpirational water loss^17^,^31^. Their form and function can be thus consistent with their interspecific variations in resource-use strategy.

The recovery of embolized vessels is critical for tree survival under drought^17^,^18^,^32^. The hysteresis between the soluble sugar content and PLC (Fig. 4) and the negative relationship between soluble sugar and starch contents (Fig. 5) imply that the embolism process triggers a conversion from starch to sugar. A low starch content in xylem sapwood will result in a delay of the recovery of vessel dysfunction^25^. If the sapwood maintains a high sugar content until the next rainfall, sugar that is injected into embolized vessels may function in the recovery of dysfunctional vessels following rainfall^23^,^24^. A decrease in respiration rates with desiccation (Fig. 2D) is often observed, reducing ATP synthesis in mitochondria^33^. Such reduced respiration rates contribute to a positive carbon balance in the plant body beyond the critical threshold for metabolic homeostasis and survival during prolonged drought^13^,^34^. Energy produced by H^+^-ATPase on the plasma membrane is used for aquaporin-dependent hydraulic permeability^35^ and for the process of refilling embolized vessels^36^,^37^. Furthermore, treatment with an H^+^-ATPase inhibitor inhibits the refilling of embolized vessels^16^. Therefore, the increase in stem respiration rates following water-supply is associated with high energy requirement for the recovery of dysfunctional vessels. These facts indicate that a decrease in stored NSCs caused by drought can accelerate drought-induced tree mortality, because of the impaired hydraulic recovery of dysfunctional vessels due to carbon shortage. Thus, carbon starvation and hydraulic failure are not exclusive hypotheses in drought-induced tree mortality processes.

A theoretical perspective suspects the different processes of tree dieback according to drought conditions, i.e., severe drought will kill trees due to hydraulic failure, whereas mild drought will kill tree due to carbon starvation^7^. It is said that hydraulic failure was the main cause of forest die-off under prolonged drought (showing without the change in NSCs contents), according to simultaneous observation of NSCs and PLC in trees of an aspen forest in Colorado^9^ and of a rainforest in Amazon^10^. On the other hand, our notion that xylem embolism (i.e., an increase in PLC) seems to trigger starch-to-sugar conversion in sapwood (Figs 4 and 5 and Table S1) implies that drought-induced hydraulic failure and its recovery processes are closely linked with carbon dynamics within xylem sapwood. Although the physiological mechanisms for drought-induced tree die-offs are still controversial, their survival and death during drought events should include both processes of hydraulic failure and carbon use in xylem (Fig. 6).

The mechanism explored in this study implies that trees may become susceptible to drought after continuous environmental stress because of a gradual decrease in the resources stored in the plant body. Even if the stress is relatively mild, the amounts of NSCs reserves in individual trees will decrease during stressful periods. In such a condition, forests will be more easily destroyed by slightly more severe future droughts under global climate change. Conversely with sufficient resources, forest die-offs are mitigated even after the sudden severe drought. The stored carbon in sapwood would contribute to the homeostasis of forest ecosystems with reducing the hydraulic failure-induced forest die-offs. Such a mechanism is responsible for ongoing cases of forest die-offs for heating and drying in urban forests^38^. Under the chronic shortage in carbon resources induced by the progressing global changes, forest ecosystem may not be able to maintain the homeostasis. Therefore, we fear more extensive and much more frequent catastrophic forest die-offs via the weakening of present forests under the condition of low carbon assimilation (Fig. 6). An understanding of the carbon dynamics within the plant body will be essential for understanding the physiological mechanisms of forest growth and die-off, and their effects on terrestrial ecosystems^39^. This knowledge could show that the coordination between carbon use and water use plays an important role on resilience and vulnerability of forest dynamics to global climate change.

## Methods

This study was conducted on the Ogasawara (Bonin) Islands in Japan (27°05′N, 142°13′E) in the northern Pacific Ocean. The mean air temperature was 23.2 °C, and the mean annual precipitation was 1,287 mm for the period from 2005 to 2014. The study year (2014) was a normal year with a relatively wet summer.

**Figure 1:**
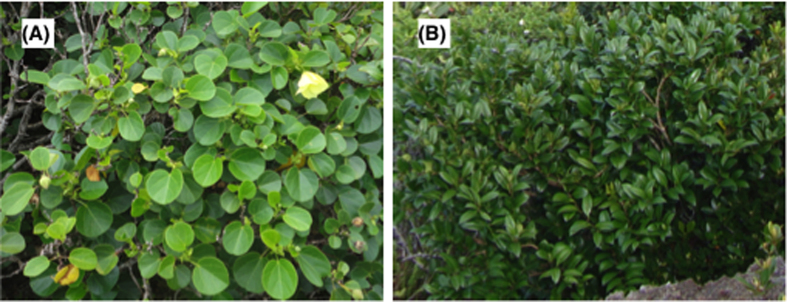
The examined adult trees growing in a dry dwarf forest in the Ogasawara Islands. (**A**) The water-spending plant, *Hibiscus glaber* Matsum. (**B**) The water-conserving plant, *Ligustrum micranthum* Zucc. *Hibiscus* trees have thinner lamina with a shorter leaf lifespan and higher leaf gas exchange rates than do the *Ligustrum* trees. The lifespan of the distal branches in *Hibiscus* is also shorter than that of the branches in *Ligustrum.*

**Figure 2:**
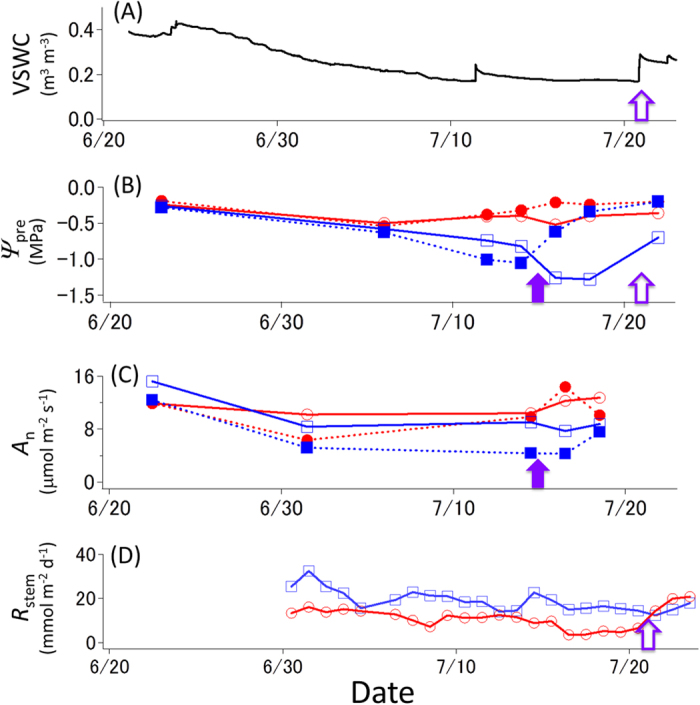
Daily time course of environmental and physiological parameters. (**A**) Volumetric soil water content (VSWC) in control trees. **(B)** Leaf water potential before dawn. **(C)** Midday net photosynthetic rates. **(D)** Daily accumulated stem respiration rates. Red circles denote *Hibiscus glaber*, and blue squares denote *Ligustrum micranthum*. Open and closed symbols denote the control and irrigated trees, respectively. For the control trees, significant rainfall occurred on 21 July (open arrow); for the irrigated trees, we supplied water to the soil on 15 and 16 July in 2014 (purple arrow).

**Figure 3:**
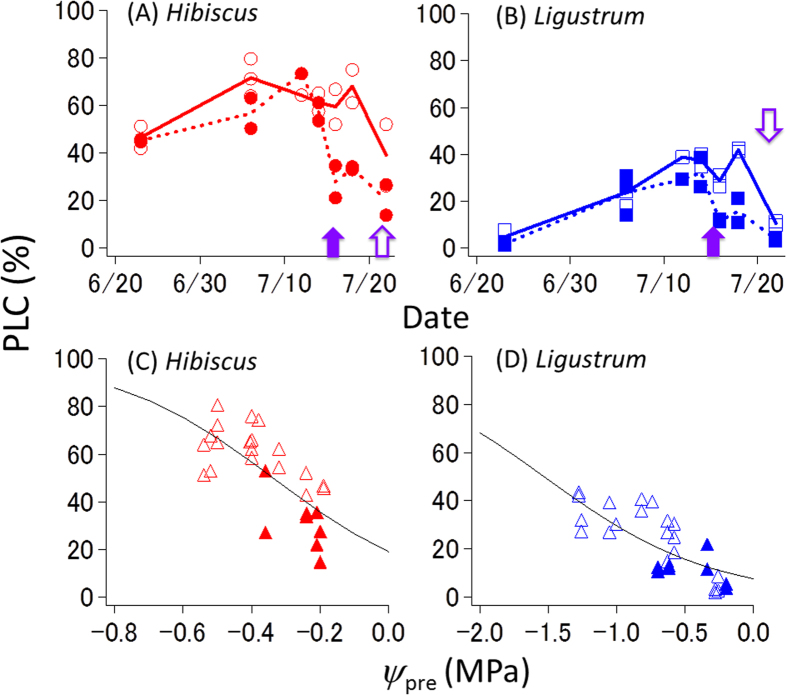
Daily time course of percent loss of conductivity (PLC) in the distal branches and the relationship between PLC and leaf water potential before dawn (*ψ*_pre_). (**A**) Daily time course of PLC in *Hibiscus glaber*. (**B**) Daily time course of PLC in *Ligustrum micranthum*. (**C**) The relationship between PLC and *ψ*_pre_ in *Hibiscus glaber*. (**D**) The relationship between PLC and *ψ*_pre_ in *Ligustrum micranthum*. In panels (**A**) and (**B**), open and closed symbols denote the control and irrigated trees, respectively. See the legend of Fig. 1 for the timing of the water supply. In panels (**C**) and (**D**), open and closed symbols show data during the dehydration process and during the rehydration process following the addition of water, respectively. The curves are fitted with a double-sigmoid function.

**Figure 4:**
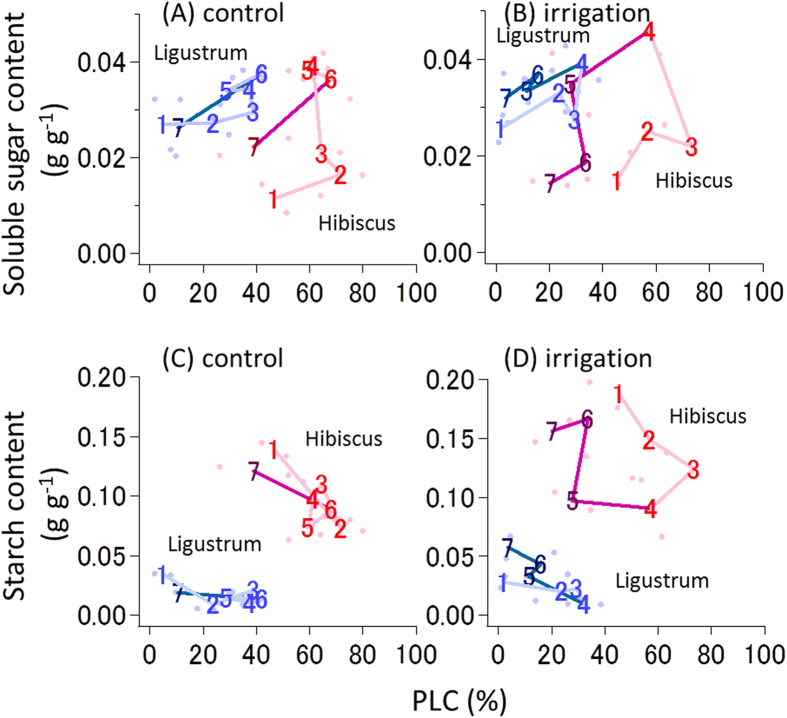
The relationship between nonstructural carbohydrates (NSCs) and PLC in the distal branches. The daily variation of soluble sugar content in the control (**A**) and irrigation trees (**B**), and that of starch content in the control (**C**) and irrigation (**D**) control trees. Red and blue colors denote *Hibiscus glaber* and *Ligustrum micranthum*, respectively. The numbers in each panel show the order of the measurement days over the experimental period from 23 June (1) to 22 July (7) in 2014. Thin lines show the period of dehydration, and thick lines show the period of rehydration after being supplied with water. See the legend of Fig. 1 for the timing of the water supply.

**Figure 5:**
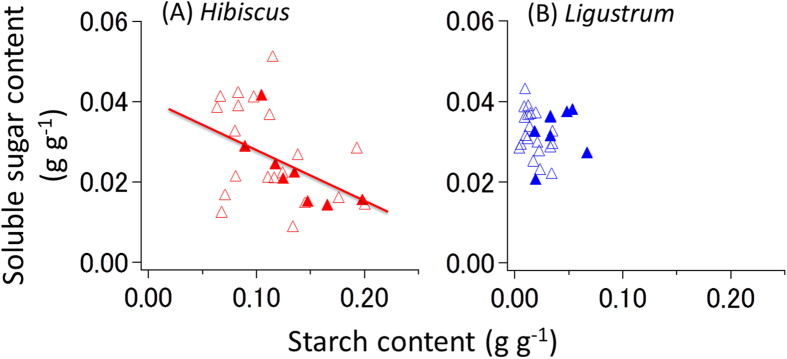
The relationship between the soluble sugar content and the starch content in the sapwood of distal branches over the experimental period. (**A**) The data show in *Hibiscus glaber*. (**B**) The data show in *Ligustrum micranthum*. Open and closed symbols show data during the dehydration process and the rehydration process after being supplied with water, respectively. A significant correlation was found in both processes in *Hibiscus* (*P* = 0.011), whereas a significant correlation was found only in the dehydration process in *Ligustrum* (*P* = 0.041).

**Figure 6:**
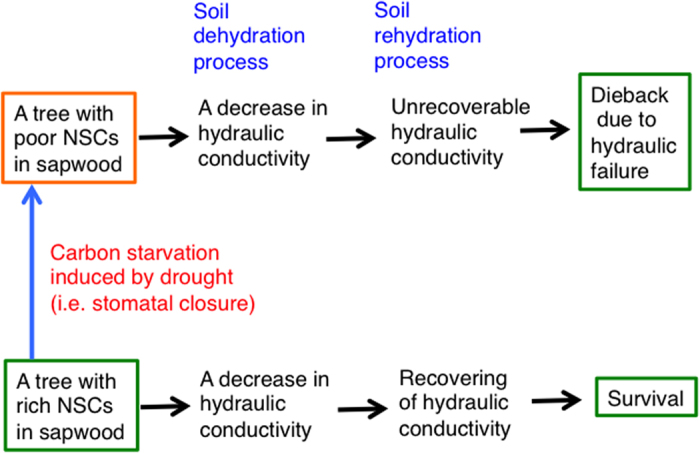
The schematic diagram of tree dieback processes. When the amount of NSCs in the sapwood decreases due to drought, xylem hydraulic dysfunction does not recover following water supply, resulting in tree dieback induced by hydraulic failure. On the other hand, when the amount of NSCs is still remained at above a threshold level during drought, xylem dysfunction is recovered following water supply, resulting in survival after the drought.

The study site is a dry dwarf forest, located on the elevated area of the island, where both *Hibiscus glaber* Matsum. and *Ligustrum micranthum* Zucc. grow sympatrically. We selected 2 healthy adult trees from each species. The heights of the canopy tops of the individuals were 1.1–1.6 m above the ground (Fig. 1). For each species, one tree was used for the artificial irrigation experiment, and the other (control) was used under natural conditions.

All measurements were performed for approximately one month, from 25 June to 22 July in 2014. The volumetric soil water content (VSWC) at a depth of 7.5 cm was monitored daily with an SM-150 (Delta-T, Cambridge, UK). For fully irrigation treatment, before sunset on 15 and 16 July, 0.6 m^3^ of water was supplied to 2.64 m^2^ of soil surface around the root systems of each individual. In natural conditions, on 21 and 22 July, increasing the VSWC in the control individuals.

Leaf gas exchange was periodically measured midday with an LI-6400 (LI-COR, Lincoln, NE, USA). Stem respiration rates at the main stem surface were measured daily by the closed-circuit method in the control individuals for each species with an infrared gas analyzer (IRGA; GMP 343, Vaisala, Helsinki, Finland). Leaf water potential before dawn was periodically measured with a pressure chamber (Model 1505-D-EXP, PMS, Corvallis, OR, USA). On the same days, 2 or 3 branches 30–50 cm in length were harvested before dawn, and twigs with 6-mm diameters, recut under water from the branches, were used for the measurements of PLC and NSCs contents. The measurement of twig hydraulic conductivity was conducted by adding gravimetric 5-kPa hydraulic pressure to the end of the examined twigs^40^. The NSCs content in the xylem sapwood was measured by quantifying the sugar content with the phenol-sulfuric acid method, and the starch content was assessed with the mutarotase-glucose oxidase method using a Glucose C-II test kit (Wako, Tokyo, Japan).

To evaluate the association between the dynamics of NSCs and water relations, the time-course changes in xylem sugar and starch contents and the amounts of xylem sugar and starch were plotted against PLC with trail of the changes. The change rates in sugar and starch contents (g g^−1^ day^−1^) were shown as:


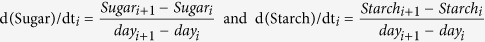


To test the contribution of water relations on the dynamics of NSCs within xylem, the relationships between NSCs content (absolute contents and change rates) and water conditions (*ψ*_pre_ and PLC) were shown with the linear regression model (see Table S1).

## Additional Information

**How to cite this article**: Yoshimura, K. *et al.* The dynamics of carbon stored in xylem sapwood to drought-induced hydraulic stress in mature trees. *Sci. Rep.* **6**, 24513; doi: 10.1038/srep24513 (2016).

## Supplementary Material

Supplementary Information
