# Supplementary material for: The dynamics of carbon stored in xylem sapwood to drought-induced hydraulic stress in mature trees
Source: Sci Rep. 2016 Apr 15;6:24513. doi: 10.1038/srep24513 (PMC4832204; doi:10.1038/srep24513)
Supplement: Supplementary Information [file srep24513-s1.doc]

**Title:** The dynamics of carbon stored within xylem sapwood to drought-induced hydraulic stress in mature trees

**Authors:** Kenichi Yoshimura^1*^, Shin-Taro Saiki^2^, Kenichi Yazaki^3^, Mayumi Y. Ogasa^3^, Makoto Shirai^4^, Takashi Nakano^5^, Jin Yoshimura^6,7,8^, Atsushi Ishida^2*^

**Author affiliations:**

^1^Kansai Research Center, Forestry and Forest Products Research Institute, Fushimi, Kyoto 612-0855, Japan.

^2^Center for Ecological Research, Kyoto University, Otsu, Shiga 520-2113, Japan.

^3^Forestry and Forest Products Research Institute, Tsukuba, Ibaraki 305-8687, Japan.

^4^Graduate School of Bioresource Sciences, Nihon University, Fujisawa, Kanagawa 252-0880, Japan.

^5^Mount Fuji Research Institute of Yamanashi Prefectural Government. Fuji-Yoshida, Yamanashi 403-0005, Japan.

^6^Department of Mathematical and Systems Engineering, Graduate School of Science and Technology, Shizuoka University, Hamamatsu, Shizuoka 432-8561, Japan.

^7^Marine Biosystems Research Center, Chiba University, Kamogawa, Chiba 299-5502, Japan.

^8^Department of Environmental and Forest Biology, State University of New York College of Environmental Science and Forestry, Syracuse, NY13210, USA.

**Table S1 The results of linear regression between non-structural carbohydrates (NSCs) and water relations.**

In the upper part, the relationships between NSCs (sugar content and starch content) within xylem and water conditions (predawn water potential and percent loss of conductivity) were shown. In the bottom part, the relationships between change rates in NSCs and water conditions. Indices of d(Sugar)/dt and d(Starch)/dt were the change rates per day in sugar and starch contents, respectively. Individuals are pooled for each species. The relationships are shown with the linear regression models (Y=*a*X+*b*).

|  |  | *Hibiscus* | | | |  | *Ligustrum* | | | |
| --- | --- | --- | --- | --- | --- | --- | --- | --- | --- | --- |
| Y | X | *a* | *b* | *R^2^* | *P* |  | *a* | *b* | *R^2^* | *P* |
| Sugar | *ψ*_pre_ | -0.028 | 0.159 | 0.094 | 0.284 |  | -0.006 | 0.028 | 0.194 | 0.115 |
| Starch | *ψ*_pre_ | 0.169 | 0.179 | 0.324 | 0.034 |  | 0.028 | 0.044 | 0.523 | 0.003 |
| Sugar | PLC | 0.018† | 0.017 | 0.073 | 0.352 |  | 0.015† | 0.028 | 0.233 | 0.081 |
| Starch | PLC | -0.112† | 0.178 | 0.273 | 0.055 |  | -0.072† | 0.040 | 0.490 | 0.005 |
|  |  |  |  |  |  |  |  |  |  |  |
| d(Sugar)/dt | *ψ*_pre_ | -0.021 | -0.008 | 0.114 | 0.283 |  | -0.015 | -0.001 | 0.035 | 0.562 |
| d(Starch)/dt | *ψ*_pre_ | 0.057 | 0.022 | 0.151 | 0.212 |  | 0.003 | 0.003 | 0.031 | 0.585 |
| d(Sugar)/dt | PLC | 0.023† | -0.012 | 0.339 | 0.047 |  | 0.008† | -0.002 | 0.104 | 0.306 |
| d(Starch)/dt | PLC | -0.048† | 0.027 | 0.254 | 0.095 |  | -0.019† | 0.006 | 0.145 | 0.223 |

†: estimated *a* values are 10^-2^ times of these values
